# The modulation of two motor behaviors by persistent sodium currents in *Xenopus laevis* tadpoles

**DOI:** 10.1152/jn.00755.2016

**Published:** 2017-03-22

**Authors:** Erik Svensson, Hugo Jeffreys, Wen-Chang Li

**Affiliations:** School of Psychology and Neuroscience, University of St Andrews, St Andrews, Fife, United Kingdom

**Keywords:** *Xenopus* tadpole, motor behavior, spinal cord, sodium currents, riluzole

## Abstract

Persistent sodium currents (*I*_NaP_) are common in neuronal circuitries and have been implicated in several diseases, such as amyotrophic lateral sclerosis (ALS) and epilepsy. However, the role of *I*_NaP_ in the regulation of specific behaviors is still poorly understood. In this study we have characterized *I*_NaP_ and investigated its role in the swimming and struggling behavior of *Xenopus* tadpoles. *I*_NaP_ was identified in three groups of neurons, namely, sensory Rohon-Beard neurons (RB neurons), descending interneurons (dINs), and non-dINs (neurons rhythmically active in swimming). All groups of neurons expressed *I*_NaP_, but the currents differed in decay time constants, amplitudes, and the membrane potential at which *I*_NaP_ peaked. Low concentrations (1 µM) of the *I*_NaP_ blocker riluzole blocked *I*_NaP_ ~30% and decreased the excitability of the three neuron groups without affecting spike amplitudes or cellular input resistances. Riluzole reduced the number of rebound spikes in dINs and depressed repetitive firing in RB neurons and non-dINs. At the behavior level, riluzole at 1 µM shortened fictive swimming episodes. It also reduced the number of action potentials neurons fired on each struggling cycle. The results show that *I*_NaP_ may play important modulatory roles in motor behaviors.

**NEW & NOTEWORTHY** We have characterized persistent sodium currents in three groups of spinal neurons and their role in shaping spiking activity in the *Xenopus* tadpole. We then attempted to evaluate the role of persistent sodium currents in regulating tadpole swimming and struggling motor outputs by using low concentrations of the persistent sodium current antagonist riluzole.

## Abstract (precis)

We have characterized persistent sodium currents in three groups of spinal neurons and their role in shaping spiking activity in the *Xenopus* tadpole. We then attempted to evaluate the role of persistent sodium currents in regulating tadpole swimming and struggling motor outputs by using low concentrations of the persistent sodium current antagonist riluzole.

transient sodium currents (*I*_NaT_) and persistent sodium currents (*I*_NaP_) are generated by the same sodium channels, depending on their opening states. *I*_NaT_ accounts for the fast depolarization during action potentials, and *I*_NaP_ have been shown to modulate spiking patterns (Bean 2007; Crill 1996; Theiss et al. 2007). Ten voltage-gated sodium channels have been identified in mammals, and six have been located in the spinal cord and/or dorsal root ganglion, where they have been implicated in pain transmission and spasticity after spinal cord injury (Brocard et al. 2016; Catterall et al. 2005; Dib-Hajj et al. 2013; Waxman and Zamponi 2014). The basic organization of the spinal locomotor circuitry and the ion channels expressed show a high degree of similarities in lower vertebrates and in mammals (Grillner and El Manira 2015; Grillner and Jessell 2009; Kiehn 2016; Roberts et al. 2012), and *I*_NaP_ has been identified in many spinal neurons (Benedetti et al. 2016; Hu et al. 2002; Miles et al. 2005; Tazerart et al. 2008; Theiss et al. 2007; Tong and McDearmid 2012; Zhong et al. 2007). A low concentration of riluzole blocks *I*_NaP_ (Urbani and Belluzzi 2000) and is used to treat amyotrophic lateral sclerosis (ALS) (Bellingham 2011; Benedetti et al. 2016; Devlin et al. 2015; Jenkins et al. 2014; Quinlan et al. 2011), which is associated with hyperactivity of spinal motor neurons and upper motor neurons in the primary motor cortex (Caballero-Hernandez et al. 2016). It is necessary to understand how *I*_NaP_ modulates motor behaviors in a trackable spinal circuit and to assess the actions of riluzole (Benedetti et al. 2016).

*Xenopus* tadpole spinal and hindbrain circuits controlling swimming and struggling have been mapped using paired whole cell recordings (Berkowitz et al. 2010; Roberts et al. 2010). Tadpole swimming central pattern generator (CPG) comprises descending interneurons (dINs), commissural interneurons (cINs), ascending interneurons (aINs), and motoneurons. Among them, dINs provide the phasic excitation to drive other types of rhythmic neurons while their own firing is sustained by rebound firing following mid-cycle inhibition or NMDA receptor (NMDAR)-dependent pacemaker properties (Li et al. 2006, Li et al. 2010; Soffe et al. 2009). When the tadpole skin is stimulated repetitively, two types of interneurons are recruited [excitatory commissural interneurons (e-cINs) and repetitive firing descending interneurons (dINrs)], but the dIN activity is suppressed (Li et al. 2007b; Li 2015). Tadpole neurons involved in swimming and struggling display different types of firing properties in response to depolarizing current pulses (Li et al. 2007a; Li et al. 2007b; Sautois et al. 2007; Winlove and Roberts 2012). dINs fire a single spike at the onset of depolarizing step currents, whereas other rhythmic neurons show repetitive firing, often with a delay caused by A-type potassium currents (Li 2015). The properties of *I*_NaT_ have been characterized in dissociated tadpole spinal neurons and sensory neurons and sensory interneurons in situ (Dale 1995; Winlove and Roberts 2012). We analyzed sodium currents in the neurons involved in tadpoles swimming and struggling in situ. We report *I*_NaP_ in the Rohon-Beard neurons (primary sensory neurons, RB), the excitatory descending interneurons (dINs), and other rhythmic neurons in tadpole swimming and struggling (non-dINs) (Roberts et al. 2008). We have used the *I*_NaP_ antagonist riluzole at 1 µM to investigate its role in tadpole swimming and struggling.

## METHODS

All experiment procedures were approved by the local Animal Welfare and Ethics Committee and comply with UK Home Office regulations. Human chorionic gonadotropin injections were carried out to induce mating between pairs of adult *Xenopus*. Tadpoles at stage 37/38 (Nieuwkoop and Faber 1956) were anesthetized using 0.1% MS-222 (3-aminobenzoic acid ester; Sigma, Irvine, UK) and then immobilized using 12.5 µM α-bungarotoxin (Tocris, Bristol, UK) and mounted onto a Sylgard stage for dissections (Moult et al. 2013). The saline contained (in mM) 127 NaCl, 3 KCl, 2 CaCl_2_, 2.4 NaHCO_3_, 1 MgCl_2_, and 10 HEPES, with pH adjusted to 7.4. Fine dissections were carried out to expose muscle clefts for recording motor nerve activities using a glass suction electrode and neuronal somata in the caudal hindbrain and rostral spinal cord for whole cell recordings (between the 5th rhombomere segments and the 7th postotic muscle segment). Intracellular signals were amplified with an Axon Multiclamp 700B, digitized with a Power 1401 mkII data acquisition interface, and sampled with Signal (version 5; CED, Cambridge, UK).

### 

#### Neuron identification and grouping.

The sensory RB neurons were initially visually identified by their large round somata and location on the dorsal edge of the spinal cord. Further RB identification was by their wide action potential and typical firing pattern (Winlove and Roberts 2012) in whole cell recordings. We grouped neurons rhythmically active during fictive swimming as dINs and non-dINs (motoneurons, commissural interneurons, ascending interneurons, repetitive firing descending interneurons), which have similar firing properties to current injections (Li et al. 2007a; Sautois et al. 2007). dINs and non-dINs were identified by their responses to light dimming, which triggers swimming activity. It is possible to distinguish dINs and non-dINs by recording extracellular action potentials with a loose-patch electrode. The dINs have monophasic action potentials, and the non-dINs have biphasic action potentials (Soffe et al. 2009). Because sensory interneurons are not active during swimming (Li et al. 2004; Li et al. 2007a; Sillar and Roberts 1988), our screening method using loose-patch recordings should have systematically excluded them.

#### Current-clamp recordings.

Whole cell recording pipettes were filled with a solution containing (in mM) 100 K-gluconate, 2 MgCl_2_, 10 EGTA, 10 HEPES, 3 Na_2_ATP, and 0.5 NaGTP with 0.1% neurobiotin (Vector Laboratories, Burlingame, CA; pH adjusted to 7.4). The inclusion of neurobiotin allowed the revealing of neuronal anatomy after whole cell recordings in some recordings (Li and Moult 2012). Current-clamp recordings of spiking properties were performed in bridge mode, and stimulations in all cases were done from membrane potential set at −60 mV by injecting slow DC currents using the Multiclamp 700B controller. Microperfusion of riluzole (Tocris) was done by positioning a glass pipette with a tip opening of ~10 µm more than 30 µm upstream to the recorded soma (Li and Moult 2012). A gentle pressure was applied inside the pipette by compressing a connected 50-ml syringe for 100 µl (~200 Pa) to eject riluzole. The pipette was moved >200 µm away from the preparation, combined with gentle suction (approximately −100 Pa) to stop gravity-driven leakage when not in use.

#### Voltage-clamp recordings of sodium currents.

After identification of neurons, the preparation was bath perfused with a solution containing (in mM) 35 NaCl, 40 NMDG, 3 KCl, 10 CaCl_2_, 2.4 NaHCO_3_, 1 MgCl_2_, 10 HEPES, 40 tetraethylammonium (TEA), 1 4-aminopyridine (4-AP), and 0.15 CdCl_2_, and pH was adjusted to 7.4. The neurons were patched with an electrode filled with a pipette solution in which 100 mM K-gluconate was replaced with 100 mM cesium methanesulfonate to block potassium channels from the inside. Liquid junction potential was 5.7 mV, calculated using the Clampex 10.2 junction potential formula. This was corrected during all recordings. Leak currents were subtracted during experiments, and serial resistance compensation was done for 70%. Serial resistances accepted for voltage-clamp recordings were between 10 and 20 MΩ.

**Fig. 1.**
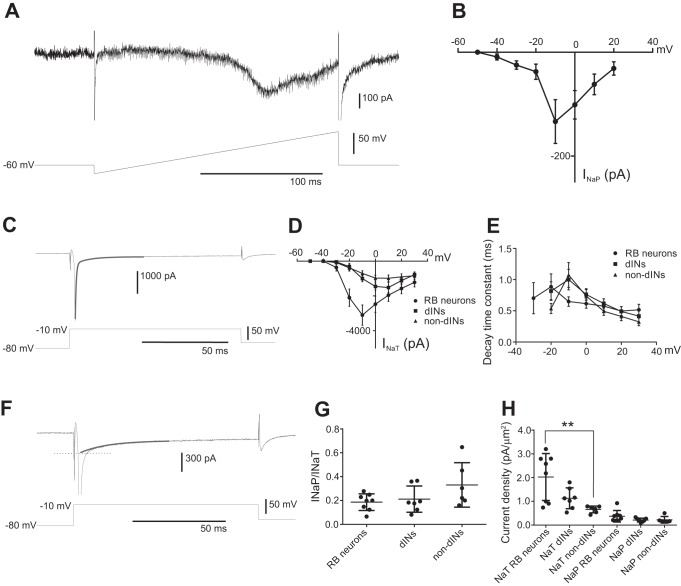
Measurements of *I*_NaT_ and *I*_NaP_ in spinal neurons. *A*: voltage ramp in an RB neuron reveals *I*_NaP_. *B*: current-voltage (*I-V*) curve of *I*_NaP_ in RB neurons measured from the ramp currents (*n* = 8). *C*: sodium currents in an RB neuron evoked by a voltage step to −10 mV. The thick gray curve shows the double-exponential fitting of the decay of the currents. *D*: *I-V* curves of *I*_NaT_ in RB neurons, dINs, and non-dINs. *E*: decay time constants of the *I*_NaT_ at steps to different voltages in the 3 neuron groups. *F*: single-exponential fitting (thick gray curve) of the slowly decaying currents. Dotted line indicates estimation of *I*_NaP_ at the peak of combined sodium currents. *G*: ratios of *I*_NaP_ to *I*_NaT_ in RB neurons, dINs, and non-dINs. *H*: current densities for *I*_NaT_ and *I*_NaP_ in RB neurons, dINs, and non-dINs. **P < 0.01.

Decay time constants were measured by double-exponential curve fitting of the recovery phase of sodium currents using the curve fitting function in Signal (Fig. 1*C*). The peak amplitude of *I*_NaP_ was measured by extrapolating a single-exponential curve fit to the slow persistent component, where *I*_NaT_ was expected to have closed (Fig. 1*F*). The surface area was calculated from the capacitance of the whole cell configuration and with the assumption that neurons had a specific capacitance of 1 µF/cm^2^ (Winlove and Roberts 2012). The current densities were then calculated by dividing *I*_Na_ (pA) with the calculated cell surface (µm^2^). Statistical significance was examined using the Kruskal-Wallis or Friedman test with Dunn’s post hoc test when the data distribution was not normal or sample size was small. When the data were normally distributed, ANOVA or Student’s *t*-tests were carried out. Means are given with SE.

## RESULTS

### 

#### I_NaP_ and I_NaT_ in tadpole spinal neurons.

Previous studies have not been able to show the presence of any *I*_NaP_ in tadpole spinal neurons (Dale 1995; Winlove and Roberts 2012). To reinvestigate this, we applied a voltage ramp (from −80 to 20 mV over 200 ms) to RB neurons. The slowly rising depolarization will inactivate the *I*_NaT_ and activate the *I*_NaP_. The ramp induced an inward current that started to open at about −40 mV and had a peak of −133.38 ± 42.12 pA at around −10 mV (*n* = 8; Fig. 1, *A* and *B*). This shows that the RB neurons possess *I*_NaP_.

To quantify *I*_NaP_ properties relative to *I*_NaT_, we used voltage steps rather than ramps in RB neurons, dINs, and non-dINs. Voltage steps ranged from −80 to 30 mV in 10-mV increments. The recovery phase of sodium currents was best fitted with a two-exponential curve, suggesting the presence of a fast-decaying (*I*_NaT_) and slowly decaying component (*I*_NaP_) (Fig. 1*C*). To estimate the size of both currents, we carried out a single-exponential fitting of the latter phase of the currents and extrapolated the size of *I*_NaP_ when the combined currents peaked (Fig. 1, *C* and *F*). *I*_NaT_ was calculated by subtracting the *I*_NaP_ component from the combined peak currents (Fig. 1, *C* and *D*). The peak *I*_NaT_ in RB neurons was −3,103.38 ± 586.25 pA (*n* = 8; Fig. 1*D*) at steps to −10 mV. The *I*_NaT_ in dINs was −1,516 ± 296.5 pA at steps to 10 mV (*n* = 7), and that in non-dINs was −979.8 ± 185.86 pA at steps to 10 mV (*n* = 6; Fig. 1*D*). The peak decay constant for *I*_NaT_ was to steps to −20 mV in RB neurons (0.89 ± 0.21 ms, *n* = 8) and at steps to −10 mV in dINs (1.00 ± 0.17 ms, *n* = 7) and non-dINs (1.08 ± 0.20 ms, *n* = 7), which is shown in Fig. 1*E*. There was no difference in the peak decay time constants for the *I*_NaT_ between neuron types (*P* > 0.05).

The ratio between the *I*_NaP_ and *I*_NaT_ amplitudes did not differ between RB neurons (0.19 ± 0.025, *n* = 8) and dINs (0.21 ± 0.042, *n* = 7; *P* > 0.05) or non-dINs (0.33 ± 0.076, *n* = 6; *P* > 0.05; Fig. 1*G*). The current density for the *I*_NaT_ was higher in 8 RB neurons (2.02 ± 0.35 pA/µm^2^) than in 7 dINs (1.13 ± 0.17 pA/µm^2^; *P* > 0.05) and 6 non-dINs (0.66 ± 0.058 pA/µm^2^; *P* < 0.01; Fig. 1*H*). There was no difference in the current density of *I*_NaP_ among the three neuron groups (RB neurons: 0.37 ± 0.086 pA/µm^2^; dINs: 0.21 ± 0.03 pA/µm^2^); non-dINs: 0.25 ± 0.084 pA/µm^2^; *P* > 0.05; Fig. 1*H*). These results show that all three neuron groups express both fast-inactivating *I*_NaT_ and a slowly inactivating *I*_NaP_.

#### Properties of I_NaP_.

**Fig. 2.**
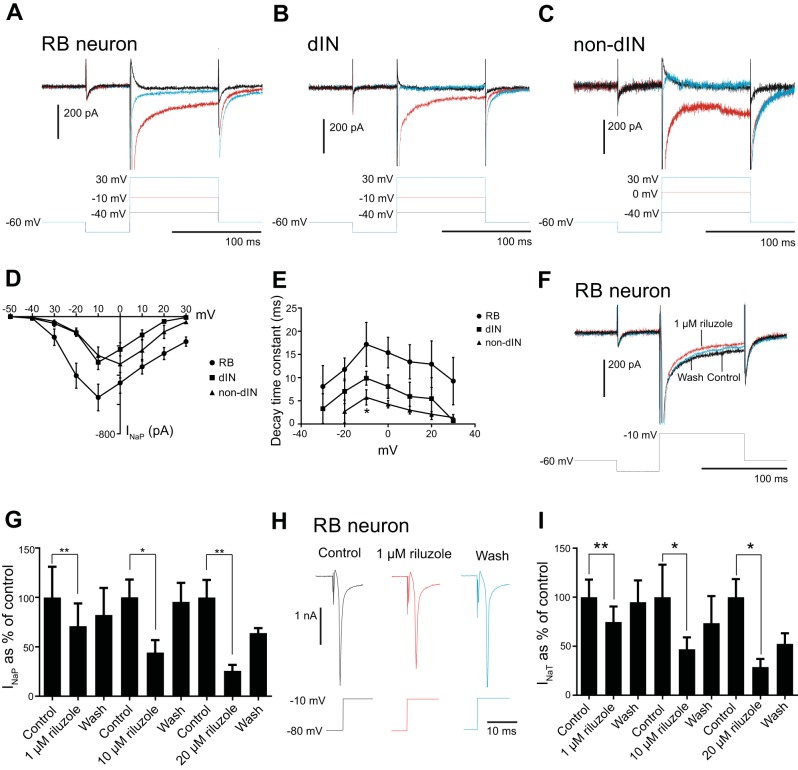
Properties of *I*_NaP_ in the 3 neuron groups and the effect of riluzole. *A–C*: sodium currents in an RB neuron (*A*), a dIN (*B*), and a non-dIN (*C*) in response to voltage step to −40 (black traces), −10, or 0 mV (red traces showing the maximal *I*_NaP_) and to 30 mV (blue traces). *D*: averaged *I-V* curves for the *I*_NaP_ in RB neurons, dINs, and non-dINs. *E*: decay time constants of *I*_NaP_ at steps to different membrane potentials in RB neurons, dINs, and non-dINs. *F*: effect of 1 µM riluzole on *I*_NaP_ in an RB neuron. *G*: blocking effects of 1, 10, and 20 µM riluzole on *I*_NaP_. *H*: effect of 1 µM riluzole on *I*_NaT_ in an RB neuron. *I*: effect of 1, 10, and 20 µM on *I*_NaT_. *P < 0.05; **P < 0.01.

We further analyzed the properties of *I*_NaP_ in these neurons on the basis of voltage-step experiments. The *I*_NaP_ in RB neurons started to activate at steps to −40 mV, and maximum current was achieved at steps to −10 mV (552.00 ± 91.17 pA; Fig. 2, *A* and *D*; *n* = 8). Steps to more depolarized voltages reduced the amplitude, and steps to 30 mV generated a current of −170.12 ± 31.52 pA (Fig. 2, *A* and *D*; *n* = 8). The decay time constant was also voltage dependent and had its maximum of 17 ± 4.7 ms at steps to −10 mV (*n* = 8; Fig. 2*E*).

Also, in the dINs the *I*_NaP_ first activated at steps to −40 mV. The maximum *I*_NaP_ was generated at steps to −10 mV (−311.43 ± 49.90 pA, *n* = 7; Fig. 2, *B* and *D*). Steps to more depolarized levels generated smaller *I*_NaP_ (Fig. 2, *B* and *D*; *n* = 7). The decay constant for the *I*_NaP_ in dINs had its peak at steps to −10 mV with a decay constant of 9.9 ± 1.5 ms (Fig. 2*E*; *n* = 7).

In the non-dINs, the *I*_NaP_ also first activated at steps to −40 mV. However, the *I*_NaP_ in the non-dINs had its peak at steps to 0 mV (−323.83 ± 94.88 pA; Fig. 2, *C* and *D*; *n* = 6). The amplitude of *I*_NaP_ decreased at more positive steps (Fig. 2, *C* and *D*; *n* = 6). The decay constant of 4.27 ± 0.80 ms at steps to 0 mV in non-dINs (*n* = 6) was faster than that in 8 RB neurons and 7 dINs (both *P* < 0.05, Student’s unpaired *t*-test; Fig. 2*E*). No difference was found between the decay time constants between RB neurons and dINs (*P* > 0.05).

Finally, we tested the action of riluzole (1, 10, and 20 µM) on *I*_NaP_ in spinal neurons. All three concentrations of riluzole significantly reduced the amplitude of *I*_NaP_ (Fig. 2*G*). A concentration of 1 µM riluzole reduced the *I*_NaP_ to 71.7 ± 15.0% of control (Fig. 2*F*; *n* = 2 dINs, 2 non-dINs, and 4 RB neurons; *P* < 0.01), 10 µM riluzole reduced *I*_NaP_ to 44.1 ± 12.7% of control (*n* = 6 RB neurons; *P* < 0.05), and 20 µM riluzole reduced *I*_NaP_ to 25.7 ± 6.0% of control (*n* = 6 RB neurons; *P* < 0.01). The effect of riluzole recovered completely or partially after washout. We also tested the action of 1, 10, and 20 µM riluzole on the *I*_NaT_. Riluzole at 1 µM reduced *I*_NaT_ significantly to 74.7 ± 15.9% of control (Fig. 2, *H* and *I*; *n* = 2 dINs, 2 non-dINs, and 3 RB neurons; *P* < 0.01). Higher doses of riluzole (10 and 20 µM) significantly reduced the amplitude of *I*_NaT_ (Fig. 2*I*). At 10 µM riluzole, *I*_NaT_ was reduced to 46.8 ± 12.2% of control (*n* = 6 RB neurons; *P* < 0.05), and at 20 µM riluzole, *I*_NaT_ was reduced to 28.8 ± 8.2% of control (*n* = 6 RB neurons; *P* < 0.05).

These results show that all three groups of neuron express *I*_NaP_, but the properties of the current vary, and 1 µM riluzole can reduce the amplitude of *I*_NaP_ and *I*_NaT_. *I*_NaP_ in non-dINs peaks at steps to 0 mV, whereas the current in RB neurons and dINs peaks at steps to −10 mV. There are also differences in the decay time constants, where non-dINs decay significantly faster than the dINs and non-dINs.

#### Effect of 1 µM riluzole on neuronal firing properties.

Riluzole is known to affect many other aspects of neuronal function, especially at concentrations much higher than 1 µM (Bellingham 2011; Urbani and Belluzzi 2000). Having demonstrated that riluzole at 1 µM could weaken *I*_NaP_, we next tested 1 µM riluzole in current-clamp mode so that we could monitor its effects on neuronal firing in the three groups of neurons. Riluzole at 1 µM did not alter the resting membrane potential, which was −62.15 mV in control, −61.32 in riluzole, and −60.77 mV after washout (*n* = 11; *P* > 0.05).

**Fig. 3.**
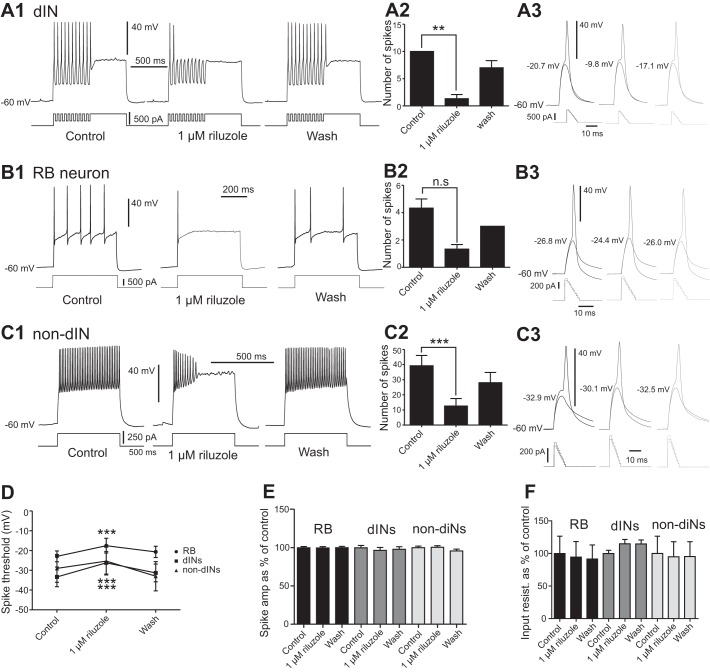
Effects of riluzole on firing properties of the 3 different neuron groups. *A1*: effect of 1 µM riluzole on the rebound firing in a dIN. *A2*: bar chart summarizing the reduction of rebound spikes in dINs (*n* = 6). *A3*: subthreshold depolarization and firing at threshold level in a dIN in control (*left*), 1 µM riluzole (**P < 0.01; *middle*), and wash (*right*). *B1*: effect of riluzole on an RB neuron with slow, repetitive firing. *B2*: bar chart showing the reduction of spikes by riluzole (*n* = 3). *B3*: subthreshold depolarization and firing at threshold level in an RB neuron in control (*left*), 1 µM riluzole (*middle*), and wash (*right*). *C1*: effect of riluzole on the repetitive spiking in a non-dIN. *C2*: reduction of number of spikes by riluzole (*n* = 9). *C3*: subthreshold depolarization and firing at threshold in a non-dIN in control (*left*), 1 µM riluzole (*middle*), and wash (*right*). *D*: effect of riluzole on the spike thresholds in RB neurons (*n* = 6), dINs (*n* = 4), and non-dINs (*n* = 5). *P < 0.05; **P < 0.01; ***P < 0.001. *E*: lack of effects of riluzole on spike amplitudes in RB neurons, dINs, and non-dINs. *F*: lack of effects of riluzole on input resistances in RB neurons, dINs, and non-dINs.

The dINs typically fire single action potentials in response to a depolarizing current pulse. However, hyperpolarizing pulses on top of depolarization to mimic inhibitory synaptic inputs generate reliable rebound spikes in dINs (Li et al. 2006) (Fig. 3*A*). Microperfusion of 1 µM riluzole reduced the number of rebound spikes from 10 in control to 1.33 ± 0.76 (from 10 hyperpolarizing pulses; Fig. 3, *A1* and *A2*; *P* < 0.001). The rebound firing recovered to 7 ± 1.32 spikes after washout (Fig. 3, *A1* and *A2*; *n* = 6; *P* < 0.01). We then tested the effect of riluzole on RB neurons with slow repetitive firing to current injections and found that the number of spikes was reduced from 4.33 ± 0.67 to 1.33 ± 0.33 (Fig. 3, *B1* and *B2*; *n* = 3; *P* > 0.05). The effect recovered partly to 3.00 ± 0.00 spikes (Fig. 3, *B1* and *B2*; *n* = 3; *P* > 0.05). Finally, we tested the effect of 1 µM riluzole on repetitive spiking in non-dINs, and the number of spikes evoked by a 500-ms depolarizing current pulse was significantly reduced from 39.22 ± 6.82 to 12.56 ± 5.55 (Fig. 3, *C1* and *C2*; *n* = 9; *P* < 0.001). This recovered to 28.00 ± 6.76 after washout.

We also monitored the effect of 1 µM riluzole on action potential amplitude, spike threshold, and input resistance in the three neuron groups. Firing thresholds were tested by injecting a 10-ms depolarizing ramp current with its peak stepped and defined as the highest depolarization before the neuron started to fire (Li 2015). In dINs, riluzole significantly increased the spike threshold from −33.35 ± 4.98 to −26.25 ± 5.67 mV (Fig. 3, *A3* and *D*; *n* = 4; *P* < 0.001) with recovery after washout of riluzole. Riluzole did not affect the spike amplitude or input resistance in dINs (Fig. 3, *A3*, *E*, and *F*; *n* = 6 for both the spike amplitude and the input resistance, *P* > 0.05). In the RB neurons, 1 µM riluzole also increased the spike threshold from −22.96 ± 2.73 to −17.55 ± 3.72 mV (Fig. 3, *B3* and *D*; *n* = 4; *P* < 0.001). The effect recovered to −20.68 ± 2.84 mV after washout (Fig. 3, *B3* and *D*). Riluzole did not change the spike amplitude or input resistance in RB neurons (Fig. 3, *B3*, *E*, and *F*; *n* = 9; *P* > 0.05). Finally, in the non-dINs, riluzole at 1 µM changed the spike threshold from −29.02 ± 7.03 to −25.34 ± 7.03 mV (Fig. 3, *C3* and *D*; *n* = 5; *P* < 0.001). The effect recovered to −33.00 ± 7.38 mV (Fig. 3, *C3* and *D*; *n* = 5). Riluzole had no effect on either the spike amplitude or input resistance in non-dINs (Fig. 3, *C3*, *E*, and *F*; *n* = 9 for spike amplitude and *n* = 7 for input resistance; *P* > 0.05).

These results show that 1 µM riluzole affects firing properties in all three groups of neurons without changing the spike amplitude or input resistance. The reduction of repetitive and rebound firing in the three groups of neuron may be due to increased firing thresholds following the partial blockade of *I*_NaP_ by riluzole.

#### Riluzole modulates swimming and struggling.

Tadpoles at stage 37/38 generate two different rhythmic motor outputs, swimming and struggling. Whereas swimming is self-sustaining after the initiation by sensory stimulation, struggling normally requires continuous activation of the mechanosensory pathway (Roberts et al. 2010). We next investigated the roles of *I*_NaP_ in these two motor patterns using 1 µM riluzole, which was shown not to have significant effect on synaptic transmission at the spinal level (Zhong et al. 2007).

**Fig. 4.**
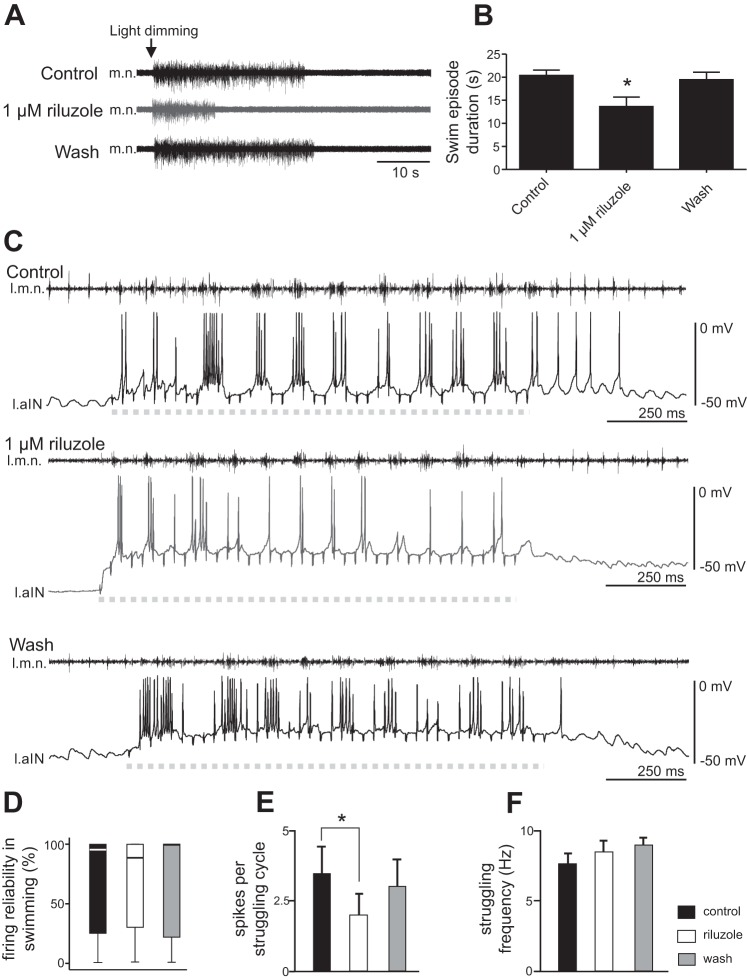
Effects of 1 µM riluzole on fictive swimming and struggling. *A*: motor nerve (m.n.) recordings during swimming evoked by light dimming in control, 1 µM riluzole, and after washout. *B*: reduction of the swimming episode duration by 1 µM riluzole (*n* = 5; *P < 0.05). *C*: effects of riluzole on the number of spikes on each struggling cycle. Dashed gray lines indicate periods of repetitive skin stimulation at the rostral trunk (40 pulses at 30 Hz), used to evoke fictive struggling. l.m.n., motor nerve recording from left side; l.aIN, ascending interneuron recording from left side. *D*: riluzole does not affect the firing reliability of neurons in swimming (% of cycles with spiking). *E*: riluzole reduces the number of spikes per struggling cycle (*P < 0.05). *F*: struggling frequencies are not affected by riluzole.

Swimming episodes in the hatching *Xenopus* tadpole can be induced by dimming the light, which activates the pineal eye on top of its forebrain (Jamieson and Roberts 2000). The lengths of swimming episodes induced by dimming the light were 20.4 ± 1.1 s in control (Fig. 4, *A* and *B*; *n* = 5). Riluzole at 1 µM was locally perfused onto the hindbrain, and this reduced the duration of swimming episodes to 13.6 ± 2.1 s (Fig. 4, *A* and *B*; *n* = 5; *P* < 0.05). The swim episode duration recovered after washout to 19.4 ± 1.6 s (Fig. 4, *A* and *B*). This result shows the *I*_NaP_ can modulate swimming episode duration. The shortening of swimming episode length could result from increased neuronal firing thresholds, or reduced neuronal excitability, in the presence of riluzole (Fig. 3*D*). We next analyzed neuronal firing reliability during swimming, defined as the percentage of cycles with neuronal spiking. However, we did not identify any change in firing reliability in 1 µM riluzole (Fig. 4*D*; *n* = 7 non-dINs; related samples Friedman’s 2-way ANOVA by ranks, *P* = 0.82).

The other motor output from the tadpole spinal/hindbrain circuit is struggling, during which neurons fire multiple spikes on each struggling cycle (Li et al. 2007b). In seven tadpoles, repetitive skin stimulation of the rostral trunk skin was used to induce fictive struggling (Li et al. 2007b). Because we have shown that riluzole application could reduce repetitive firing of non-dINs at rest, we analyzed whether riluzole could weaken non-dIN firing during struggling. The struggling rhythms became irregular in two tadpoles when 1 µM riluzole was bath applied. In the other five tadpoles, fictive struggling persisted throughout riluzole application (Fig. 4*C*). There was a decrease in the number of spikes per struggling cycle (*n* = 5 non-dINs; *P* < 0.05, paired *t*-test; Fig. 4*E*). During struggling with higher frequencies, there can be fewer spikes on each cycle because burst duration is shorter. However, there was no change in struggling frequencies in the presence of riluzole (Fig. 4*F*), suggesting the reduction of neuronal spiking during struggling may be a direct effect of 1 µM riluzole on the recorded neuron.

## DISCUSSION

### 

#### I_NaP_ in spinal/hindbrain circuits.

*I*_NaP_ has been widely identified in spinal circuits controlling locomotion (Dai and Jordan 2011; Heckman et al. 2008; Tazerart et al. 2007; Theiss et al. 2007; Zhong et al. 2007; Ziskind-Conhaim et al. 2008) and also in preBötzinger complex neurons critical for generating breathing rhythms (Del Negro et al. 2002; Del Negro et al. 2005; Koizumi and Smith 2008; Pace et al. 2007). We have identified *I*_NaP_ in the sensory RB neurons and in all groups of neurons involved in rhythmic swimming and struggling activity. The majority of *I*_NaP_, with other persistent inward currents, may be located on dendrites (Hounsgaard and Kiehn 1993; Lee and Heckman 2000; 1996), whereas others have reported the generation of *I*_NaP_ in the proximal axon (Astman et al. 2006; Osorio et al. 2010). This may explain why clear *I*_NaP_ was not recorded in the isolated *Xenopus* tadpole spinal neurons (Dale 1995), where neurons lose axons and most dendrites during the dissociation process. This study also shows that the activation of *I*_NaP_ will require stepping the membrane potential above −40 mV, peaking at around 0 mV. The absence of *I*_NaP_ in the RB neurons in a previous study may be a result of using depolarizing voltage steps below −40 mV (Winlove and Roberts 2012).

#### Role of I_NaP_ in regulating spiking activity.

In this study we show that three groups of neuron in the tadpole display *I*_NaP_ but that the properties of the current differ slightly between the neuron types. The *I*_NaP_ in all neuron groups started to be activated at voltage steps to −40 mV, which is below the threshold for the action potential in all three neuron types (Figs. 1*A*, 1*B*, 2*E*, and 3*D*) (Li 2015). This suggests that depolarization from synaptic currents or current injections may just need to depolarize the membrane potential to the level to activate *I*_NaP_. The activation of *I*_NaP_ then will depolarize the membrane potential further to trigger spiking. Therefore, *I*_NaP_ can play a role in setting the spike threshold, defined as the highest depolarization before spiking in this study. Indeed, increased *I*_NaP_ current density is correlated with more negative spike threshold (Bellingham 2013; Kuo et al. 2006). This was supported by the observation that riluzole reduced the amplitude of *I*_NaP_ and significantly decreased the excitability of all three groups of neurons. One difference between the *I*_NaP_ in the three neuron groups was that *I*_NaP_ peaked at voltage steps to −10 mV in RB neurons and dINs, whereas the peak in non-dINs was at steps to 0 mV. The decay time constant also differed in that RB neurons had the slowest decay and non-dIN I_NaP_ decayed the fastest. We do not know how much these differences can be explained by the space-clamping issues, which depend on the anatomy of neurons and the distribution of ion channels. One explanation can be that the different groups of neurons express different type of sodium channel isoforms. Non-dINs in the tadpole spinal cord and hindbrain typically show repetitive firing to depolarizing current injections, and they have a higher *I*_NaP_/*I*_NaT_ ratio than the dINs and RB neurons (Fig. 3, *C1* and *C2*) and the fastest *I*_NaP_ decay time constant. These properties of *I*_NaP_ may help to shape their repetitive firing, as suggested in the 11- to 19-day-old rat ventral horn interneurons in the lumbar cord region (Theiss et al. 2007). The potential of *I*_NaP_ in setting neuronal firing thresholds has also been reported in the commissural interneurons and motoneurons of neonatal mouse spinal cord (Zhong et al. 2007).

#### Role of I_NaP_ in regulating tadpole swimming and struggling.

*I*_NaP_ is not thought to be essential in the generation of respiratory-related rhythm because riluzole microinjection in the mouse preBötzinger complex did not perturb respiratory frequency in a slice preparation (Pace et al. 2007), although *I*_NaP_ plays a role in the pacemaker bursting of some neurons therein (Del Negro et al. 2002; Koizumi and Smith 2008). *I*_NaP_ also contributes to the induced oscillations in some rodent spinal interneurons (Bouhadfane et al. 2013; Tazerart et al. 2008; Ziskind-Conhaim et al. 2008). *I*_NaP_ may help to stabilize the fictive locomotion rhythms in neonatal rats (Tazerart et al. 2007) and is suggested to have an essential role in the generation of neonatal mouse locomotion pattern (Zhong et al. 2007) or in generating the rat masticatory movements (Brocard et al. 2006).

Does *I*_NaP_ play a role in the generation of tadpole swimming rhythms? The generation of swimming rhythms relies on rebound/pacemaker firing in dINs (Li 2011). In this report we have shown that riluzole shortened swimming episodes. It is known that tonic depolarization in dINs during swimming is needed in dIN rebound firing (Li and Moult 2012). *I*_NaP_ has been suggested to amplify excitatory synaptic inputs and prolong membrane depolarization and firing (Lee and Heckman 1996). The activation of *I*_NaP_ can enhance NMDAR-mediated depolarization in dINs during normal swimming and contribute to the maintenance of swimming rhythms. dINs in tadpoles also show large membrane oscillations when their NMDARs are activated (Li et al. 2010). The activation threshold for *I*_NaP_ is within the oscillation voltage range, suggesting *I*_NaP_ should play a role in the NMDAR-dependent oscillations, although further direct experiments on *I*_NaP_ and NMDAR interactions are needed. Furthermore, the activation of *I*_NaP_ in dINs at membrane potentials below their firing threshold may facilitate their rebound firing following inhibition. Indeed, our results show riluzole has decreased dIN rebound firing reliability (Fig. 3, *A1* and *A2*). However, the firing reliability of rhythmic neurons during swimming is not affected by 1 µM riluzole. Firing of dINs as a cell group during swimming is very robust due to the extensive electrical coupling among them (Li et al. 2009). Reliable dIN firing can in turn sustain non-dIN firing on each swimming cycle (Soffe et al. 2009). Because the brain stem dINs play critical roles in the maintenance of tadpole swimming, the shortening of swimming may be a result of reduced activity in a small number of dINs, which can alter swimming activity (Moult et al. 2013) but missed in our recordings. Another confounding factor is that whole cell recordings can alter the cytoplasmic ionic composition and lead to rundown of certain ion channel currents, which may cause mismatching changes in neuronal spiking in whole cell recordings and network activities such as swimming. We also cannot exclude the possibility that swimming is shortened due to the presence of some subtle, unobserved effects from riluzole application.

Tadpole struggling rhythms involve high-frequency repetitive firing of spinal and hindbrain neurons (non-dINs), whereas dINs appear to play a minor role because their firing is weak (Li 2015; Li et al. 2007a). *I*_NaP_ has been shown previously to promote repetitive firing in rat ventral horn neurons (Theiss et al. 2007a). Riluzole has similar effect on non-dIN repetitive firing evoked either by current injection (Fig. 3*C*) or during struggling (Fig. 4, *C* and *E*). Therefore, the depression of non-dIN repetitive firing by riluzole should affect the generation of struggling rhythms. Meanwhile, RB neurons need to be activated repetitively to evoke struggling rhythms (during repetitive skin stimulation), and they can be another target for riluzole. Riluzole reduced the number of spikes CPG neurons fire on each struggling cycle. This may result indirectly from weakened RB outputs to the CPG circuit and directly from suppression of CPG excitability by riluzole. However, the frequency of struggling rhythms was not affected by riluzole at 1 µM (Fig. 4*F*), although we still do not fully understand what controls struggling frequencies. The lack of effect could be due to the limited block of *I*_NaP_ by riluzole at 1 µM. Increasing riluzole concentration will affect chemical synaptic transmission (Bellingham 2011; Mohammadi et al. 2001), calcium (Huang et al. 1997), potassium (Cao et al. 2002), and transient sodium channels (Kuo et al. 2006) and make the results very difficult to interpret. Therefore, we do not know if swimming or struggling rhythms could persist through a full blockade of *I*_NaP_ unless a more specific way to block *I*_NaP_ becomes available.

In this study, we have revealed the wide expression of *I*_NaP_ in all groups of tadpole spinal neurons. Although we have reported some effects of 1 µM riluzole on tadpole swimming and struggling behavior, it is difficult to evaluate how *I*_NaP_ shapes the swimming and struggling outputs because of the lack of specificity of riluzole blockade of *I*_NaP_ and its indiscriminate targeting of all neuronal types with *I*_NaP_ expression. To genetically modify *I*_NaP_ currents targeting specific types of neurons in the motor rhythm generation circuits may provide more definitive insights into the *I*_NaP_ modulation of motor outputs (Brocard et al. 2016).

## GRANTS

This work was financially supported by Biotechnology and Biological Sciences Research Council Grant BB/L00111X/1.

## DISCLOSURES

No conflicts of interest, financial or otherwise, are declared by the authors.

## AUTHOR CONTRIBUTIONS

E.S., H.J., and W.-C.L. performed experiments; E.S., H.J., and W.-C.L. analyzed data; E.S., H.J., and W.-C.L. interpreted results of experiments; E.S., H.J., and W.-C.L. prepared figures; E.S. drafted manuscript; E.S. and W.-C.L. edited and revised manuscript; E.S., H.J., and W.-C.L. approved final version of manuscript.
